# Predictors of mortality and ICD shock therapy in primary prophylactic ICD patients—A systematic review and meta-analysis

**DOI:** 10.1371/journal.pone.0186387

**Published:** 2017-10-17

**Authors:** Leonard Bergau, Tobias Tichelbäcker, Barbora Kessel, Lars Lüthje, Thomas H. Fischer, Tim Friede, Markus Zabel

**Affiliations:** 1 Department of Cardiology and Pneumology, University Medical Center Göttingen, Göttingen, Germany; 2 Department of Medical Statistics, University Medical Center Göttingen, Göttingen, Germany; 3 DZHK (German Center for Cardiovascular Research), partner site Göttingen, Göttingen, Germany; Kurume University School of Medicine, JAPAN

## Abstract

**Background:**

There is evidence that the benefit of a primary prophylactic ICD therapy is not equal in all patients.

**Purpose:**

To evaluate risk factors of appropriate shocks and all- cause mortality in patients with a primary prophylactic ICD regarding contemporary studies.

**Data source:**

PubMed, LIVIVO, Cochrane CENTRAL between 2010 and 2016.

**Study selection:**

Studies were eligible if at least one of the endpoints of interest were reported.

**Data extraction:**

All abstracts were independently reviewed by at least two authors. The full text of all selected studies was then analysed in detail.

**Data synthesis:**

Our search strategy retrieved 608 abstracts. After exclusion of unsuitable studies, 36 papers with a total patient number of 47282 were included in our analysis. All-cause mortality was significantly associated with increasing age (HR 1.41, CI 1.29–1.53), left ventricular function (LVEF; HR 1.21, CI 1.14–1.29), ischemic cardiomyopathy (ICM; HR 1.37, CI 1.14–1.66) and co-morbidities such as impaired renal function (HR 2.30, CI 1.97–2.69). Although, younger age (HR 0.96, CI 0.85–1.09), impaired LVEF (HR 1.26, CI 0.89–1.78) and ischemic cardiomyopathy (HR 2.22, CI 0.83–5.93) were associated with a higher risk of appropriate shocks, none of these factors reached statistical significance.

**Limitations:**

Individual patient data were not available for most studies.

**Conclusion:**

In this meta-analysis of contemporary clinical studies, all-cause mortality is predicted by a variety of clinical characteristics including LVEF. On the other hand, the risk of appropriate shocks might be associated with impaired LVEF and ischemic cardiomyopathy. Further prospective studies are required to verify risk factors for appropriate shocks other than LVEF to help select appropriate patients for primary prophylactic ICD-therapy.

## 1 Introduction

Implantable cardioverter defibrillators (ICDs) are established as the standard therapy in patients at risk of sudden cardiac death (SCD). Since the early 2000s, patients with an impaired left ventricular function (LVEF) of ≤35%, either due to ischemic (ICM) or dilated cardiomyopathy (DCM), are routinely provided an ICD for primary prophylaxis (PP) of SCD. [1],[2–4]. To date, this indication applies to the majority of ICD implantations in Europe[5]. More than a decade after publication of the MADIT- II and SCD-HeFT trials, evidence is mounting that the benefit of ICD therapy is not equal in all patients with a primary prophylactic indication.

Koller et al., for instance, demonstrated that a substantial part of ICD patients decease without requiring ICD therapy prior to death[6]. In concordance, a recent registry published by Kramer et al.[7] reported an average annual mortality rate of 5.2%, however, with a wide mortality range between 1.7% and 18.2%, depending naturally on age and on few clinical criteria including creatinine level, left ventricular ejection fraction [LVEF], peripheral arterial disease. Similar results were confirmed by Barsheshet et al [8] and van Welsenes et al [9].

Currently, existing meta-analyses that focused on the benefit of primary prophylactic ICD treatment, have all featured predominantly relatively old studies including randomized controlled trials such as MADIT and SCD-HeFT[10,11]. Therefore, there is a need for quantitative synthesis of evidence arising from more recent studies. With regard to gender Conen et al^12^ very recently closed this gap by conducting a systematic review and meta-analysis of studies published since 2010 and reporting the prognostic effects of gender on risk of appropriate ICD therapy and all-cause mortality in primary prophylactic ICD-patients. This systematic review and meta-analysis aims to complement the work by Conen et al by considering prognostic factors other than gender for the risk of appropriate ICD therapy and all-cause mortality in primary prophylactic ICD-patients.

## 2 Methods

The applied methodology follows broadly the lines of the systematic review and meta-analysis presented in Conen et al[12]. Furthermore the same literature search as in Conen et al underlies the results herein; this will be explained in more detail below.

### Search strategy

We searched PubMed, LIVIVO (provided by the publicly funded ZB MED Information Centre for Life Sciences in Cologne, Germany) and Cochrane CENTRAL (date of last search: May 11, 2016) for relevant publications from 2010 and later using the following search terms: ("primary prophylaxis" OR "primary prophylactic" OR "primary prevention") AND ("ICD" OR "defibrillator") AND ("mortality" OR "shock" OR "death" OR "ICD therapy" OR "ICD treatment") without any language restrictions. The limitation on the publication year was chosen to focus the search on studies that had enrolled predominantly primary prophylactic ICD patients after the publication of the major landmark trials and corresponding guidelines in this field. The outcome of our search strategy was checked against a pre-defined list of 19 publications that are related to the topic and that had been compiled prior to the search. In addition, reference lists of the selected papers were screened for further relevant publications.

### Study selection

Two authors (LB, BK) reviewed the abstracts identified by the literature search. If an abstract was judged as potentially relevant by at least one of the reviewers, the full-text of the publication was screened for

1. appearance of at least one of the three end-points of interest: first appropriate shock, first inappropriate shock or all-cause mortality
2. reported factor-specific effects on at least one of the end-points.

The factors we concentrated on were: age at implantation, LVEF, NYHA class, cardiac resynchronization therapy with ICD, comorbidities (atrial fibrillation, diabetes mellitus, ischemic cardiomyopathy), renal function and prescribed medication (Amiodarone, beta-blockers, diuretics). Note that gender effects were examined previously and reported by Conen et al.

Furthermore, we required the reported effect to be observed among patients with an implanted ICD, possibly (but not exclusively) with cardiac resynchronization therapy (CRT), and who were enrolled at the time of device implantation, in order to avoid survival bias. To focus our results on patients with an ICD implanted for primary prevention, we considered only papers in which at least 60% of the study population received a primary prophylaxis ICD, or in which results for the primary prevention subgroup were reported separately.

In two studies, we had access to the individual patient data and repeated the original data analysis within the primary prevention subgroup. Papers considering very specific patient populations, e.g. all patients older than 80 years, patients after CABG surgery only or all patients on dialysis, were excluded.

### Assessment of the risk of bias

Our search identified results predominantly from observational studies, therefore we assessed the risk of bias in this systematic review by focusing on three domains (selection of participants, measurement of variables and outcomes, control of confounding) as previously recommended[13]. Low risk of bias would be associated with the inclusion of all consecutive patients, with a description of the determination of outcomes and details about measurement and definition of other variables, and with accounting for confounders in the analysis. We also looked at the reporting of missing values and its handling throughout the analysis. The study quality was assessed by two reviewers (BK, LB).

### Data extraction

Hazard ratios measuring the effect of the investigated factors were chosen as the measure of interest. We extracted point estimates from univariable or multivariable proportional hazards models, together with their standard errors on the log hazard scale (when available) and associated confidence intervals (CI). The texts were screened for consistent reporting of the results, in order to avoid possible typographical errors. For studies in mixed populations of primary and secondary prevention patients where we had access to the individual patient data, we repeated the analyses in the primary prevention subgroup, if such results were not reported in the original papers. Note that in case of the first appropriate shock there is a competing terminal event (death) precluding observation of the endpoint of interest. In such a competing-risk situation, the hazard ratios can be obtained using either a Cox proportional hazards model, or a Fine & Gray model[14]. The interpretation of the hazard ratios from the two models differs. The reason is that with competing risks, the instantaneous risk of the event of interest does not fully determine the cumulative incidence of the event. The cumulative incidence of first appropriate shocks depends both on the instantaneous risk of the shock as well as on the instantaneous risk of death. The hazard ratios coming from the Cox proportional hazards model are related to the instantaneous risks of the first appropriate shock (and on their own do not allow for making conclusions about cumulative incidences). On the other hand, hazard ratios in the Fine & Gray model are directly related to the cumulative incidence of the first appropriate shocks. However, they do not say anything about instantaneous risks.

In addition, the following study characteristics were extracted: the total number of patients included in the analysis, duration of follow up, years of ICD implantation, composition of the study population with respect to gender, age, ischemic cardiomyopathy, NYHA functional class, left ventricular ejection fraction (LVEF), diabetes mellitus, cardiac resynchronization therapy and primary versus secondary prevention. The data extracted by one author (BK) were independently verified by another author (LB).

### Statistical analysis

After extraction, the hazard ratios (HR) were log-transformed and their standard errors, if not available directly, were calculated from the reported 95% CIs (assuming usage of normal quantiles). Hazard ratios were combined in random effects meta-analyses with inverse variance weighting, i.e. allocating larger weights to more precise studies. In the primary analysis adjusted and unadjusted HR were considered. Additionally we performed a sensitivity analysis by using solely multivariate HR. Since in several cases only few studies were available reporting appropriate hazard ratios, the pooling was done employing the Bayesian approach as recommended[15,16] using half-normal prior with scale parameter 0.5 for the between-study standard deviation and uniform prior for the pooled effect. Sensitivity analyses were conducted by considering two additional priors for the between-study standard deviation: the prior recently suggested by Bodnar et al.[17] and the half-normal prior with scale parameter 1[15]. The results of the sensitivity analysis are reported only when discrepancies arose. We report the posterior median of the pooled effect and the shortest 95% credible interval for it as implemented in the R package bayesmeta[18]. We also report the posterior median and the shortest 95% credible interval for the between-study standard deviation, which reflects the between-study heterogeneity. The Bayesian approach updates the prior information on the plausibility of different values of the pooled effect and the between study standard deviation with the data, yielding a posterior distribution for the two parameters. Note that our choice of the prior distribution for the pooled effect is noninformative, giving the same weight to any value.

For comparison, in all cases when meta-analysis is performed, we also show the pooled effect with 95% confidence interval as obtained using normal quantiles and the DerSimonian-Laird estimator for the between-study variance as this is still the standard approach for random effects meta-analysis in some standard software such as the Cochrane Collaboration’s software RevMan. Nevertheless, this procedure has been observed to yield too optimistic confidence intervals in some settings[16,17]. The calculations were done using the R package metafor[19].

All analyses were done using the R software (R Foundation for Statistical Computing, Vienna, Austria). We judged “significance” by considering the credible intervals.

## 3 Results

### Search results

**Fig 1:**
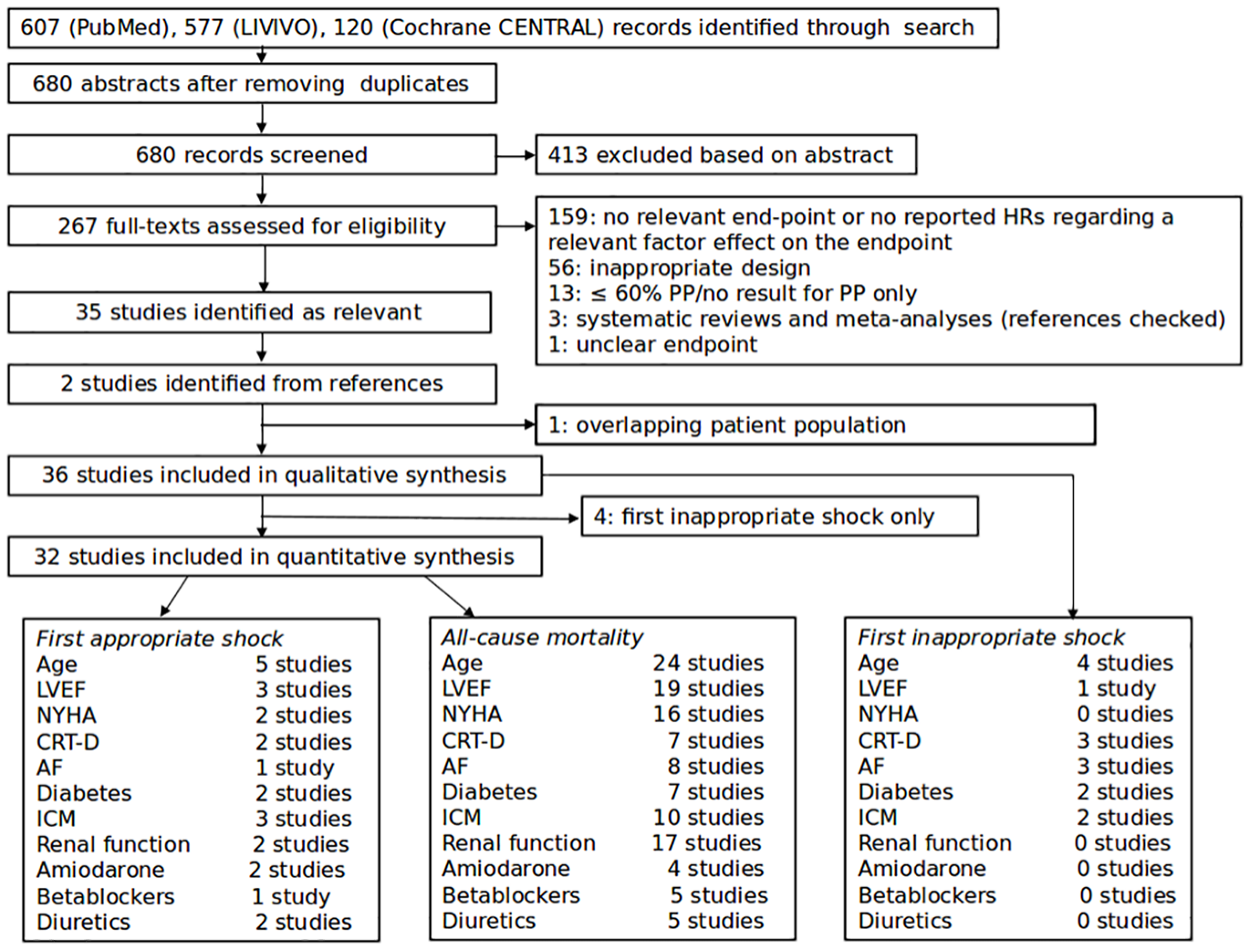
Flow chart showing the results of the literature search and the number of available studies regarding the chosen end-points and the considered factors. HR stands for hazard ratio and PP for primary prevention.

Our search strategy identified 680 abstracts (after removing duplicates), out of which 267 were selected for full-text screening. After the assessment for eligibility, 159 full-texts were excluded, since they did not report hazard ratios regarding a relevant factor effect on one of the relevant end-points, 56 were judged unsuitable based on their design and further 17 full-texts were excluded for reasons shown in Fig 1. This left us with 35 papers, whose references were checked and 2 additional full-texts were identified as fulfilling our criteria. Furthermore, we excluded Schmidt et al.[20] from the quantitative synthesis because the studied population overlapped to a large extent with that in Weeke et al.[21] and the latter work used a more precise definition of the primary prevention group. Out of the remaining 36 published papers, 3 pairs[22–27] analysed the same registries. However, the models used and the factors considered differed partially (in the latter two cases the selection of patients for the analysis differed as well), what prevented us from choosing one from each pair over the other for our analysis. Furthermore, there appeared to be a large overlap in patient populations in Fernandez-Cisnal et al.[28] and Rodriguez-Manero et al.[29], and a small part of patients analysed by Hage et al.[30], Hager et al.[31], Levine et al.[32], Masoudi et al.[33], Raja et al.[34] might be a part of the large populations taken from the American NCDR ICD registry as analysed by Bilchick et al.[35] and Hess et al.[36]. A total of 36 studies were included in the qualitative synthesis. However, due to insufficient data the endpoint time-to-inappropriate shocks was not considered for quantitative synthesis. Since 4 studies reported data on only the first inappropriate shock(Biton et al, Chen et al, Fernandez-Cisnal et al, Kutyifa et al.), the number of studies included in the quantitative analyses is 32. Disregarding these overlaps, Fig 1 shows an overview of the number of studies available for the different end-points and the considered factors.

### Assessment of bias

The 36 studies identified as relevant by our search and included in the qualitative analyses were mostly observational studies recruiting consecutive patients undergoing ICD implantations. Five studies[37–41] reported secondary analyses of the MADIT-RIT trial, which was a randomized controlled trial comparing three programming strategies. In 5 studies, the consecutive patients were further restricted: to patients without prior atrial fibrillation[42,43] to only males with GGT-levels available[44], to patients with NT-proBNP or BNP levels measured and a parallel cohort without these levels[32] and to patients with coronary angiography available[34]. All the studies described the inclusion/exclusion criteria used to define their cohorts and these matched our objectives. Table 1 summarizes the baseline characteristics of the patient populations over the studies.

**Table 1:** Summary of baseline characteristics of the patient populations (altogether 31 patient populations were considered, after counting the doubly analysed French Registry[22,23] and the 5-times analysed MADIT-RIT population[37–41] only once).

Description of the characteristic	Number of evaluated patient populations (max. 31)	Median, (min–max)	% of patient populations with the characteristic
Number of patients	31	632, (94–47282)	
Start of the recruitment [year]	30	2003, (1992–2010)	
Recruitment started before 2002	30		20%
Single-centre population	30		47%
Multi-centre population (more than 2 centres)	30		40%
Length of follow-up in years (mean or median)	28	2.7, (0.9–5.4)	
Only primary prevention patients	31		84%
Females (%)	30	17%, (0% -30%)	
Age (mean or median)	27	65, (58–69)	
CRT-D (%)	23	37%, (0%-65%)	
ICM (%)	27	69%, (53%–100%)	
NYHA III or IV (%)	25	39%, (14%–74%)	
LVEF (mean or median) in %	22	27, (24–33)	
Diabetes (%)	24	32%, (17%–55%)	

All 36 studies described the determination of outcomes and the majority (n = 21) provided details regarding the measurement of at least some of the covariates considered. In all studies, the statistical analysis accounted for confounders in a certain way. In some cases, however, only results coming from univariable models were available when specific factors were of our interest. Such results are marked with * in our analysis. Table 2 shows the overview of the considered confounders. The reporting regarding missing values and their handling in the analysis was very poor. Only 6 studies[25,31,33,36,45] clearly stated the presence/absence of missing values and the approach to them in the analysis. Especially the latter was vastly omitted in reporting.

**Table 2:** Overview of covariates employed in multivariable models in the different studies. Hazard ratios from these models (if not stated otherwise) entered our analysis and are reported throughout this paper. M stands for all-cause mortality, AS for the first appropriate shock.

Study	End-point	Covariates in the model
Bilchick et al[35]	M	Abbreviated model (used for Age, NYHA, LVEF): Age, NYHA, LVEF, AF, diabetes, CKD, COPD
		Full Model: Age, gender, race, QRS, AF, bundle branch block, LVEF, NYHA, duration of HF, diabetes mellitus, COPD, CKD, prior myocardial infarction, prior CABG, systolic BP, diastolic BP, heart rate, digoxin, beta-blockers, ACE inhibitors, diuretic agents, Amiodarone, Warfarin, breast cancer, colon cancer, prostate cancer, depression
Campbell et al[42]	M	Renal impairment, ethnic origin
Demirel et al[46]	M	Age, LVEF
Dichtl et al[44]	M	ICM, LVEF, GFR, Age, AF, QRS, NYHA, beta-blockers, Amiodarone, GGT level
Fauchier et al[23]	M	Age, NYHA, LVEF, early complication, AF, coronary artery disease, gender, QRS, eGFR, number of comorbidities, history of stroke, chronic lung disease, cancer, diabetes, type of device, Amiodarone, antiplatelet therapy, ACEi/ARB-II, beta-blockers, oral anticoagulation, sotalol, spironolactone
Gatzoulis et al[47]	M	Age, gender, LVEF, NYHA, ischemic cardiomyopathy, secondary prevention
	AS	Age, gender, ischemic cardiomyopathy, secondary prevention
Gigli et al[45]	M	Age, gender, ischemic cardiomyopathy, single-/dual-chamber ICD, LVEF
Hage et al	M	Age, gender, hypertension, atrial fibrillation, myocardial infarction, CKD, LVEF, left bundle branch block, biventricular pacing, anti-arrhythmics (including, but not limited to amiodarone and beta-blockers)
Hager et al[31]	M	Age, CKD, diabetes mellitus, peripheral arterial disease, ejection fraction
Konstantino et al, IMAJ[26]	M	Age, LVEF, chronic renal failure, implantation indication
Konstantino et al, ICE[43]	M	Age, diabetes mellitus, single-/dual-chamber ICD, NYHA, LVEF
Kraaier et al[48]	M	Model (used for age, AF, LVEF, renal function): Age, COPD, history of AF, LVEF, QRS, eGFR, NYHA, type of device
Lee et al[25]	AS	Model (used for age, AF, Amiodarone, renal function): Age, sex, nonsustained VT, atrial fibrillation, pre-existing pacemaker system, smoker, digoxin, Amiodarone, creatinine, haemoglobin, QRS
Levine et al[32]	M	History of appropriate ICD therapy, sex, age, LVEF, NYHA, history of CAD, BUN, atrial fibrillation, Amiodarone
Maciag et al[49]	M	Model (used for age, NYHA): Age, NYHA, previous revascularization
Masoudi et al	M	Age, LVEF, ischemic cardiomyopathy, NYHA, BUN, atrial fibrillation, diabetes, hypertension, chronic lung disease, haemoglobin, QRS, device type, ACE/ARB, betablockers
Ng et al[50]	M	Diabetes, NYHA, peri-infarct zone longitudinal strain, eGFR, CRT-D
Nombela-Franco et al[51]	M	Age, NYHA, beta-blockers, chronic total coronary occlusion
Providência et al[22]	M	Sex, atrial fibrillation, NYHA, LVEF, ischemic heart disease, GFR, QRS, CRT-D, beta-blockers, amiodarone, sprironolactone, calcium channel blockers, antiplatelet agents, vitamin K antagonists
Rodriguez-Mañero et al[29]	M	Age, sex, LVEF, creatinine, COPD, digoxin (for ICM also ICM added to the model)
Ruwald et al[39]	AS	Programming arm, previous atrial arrhythmias, age, systolic BP, LVEF, diabetes
Sedláček et al[40]	M	Age, diabetes, ejection fraction, diastolic BP, CRT-D, NYHA, ischemic cardiomyopathy
Seegers et al[52]	AS	Age, gender, Amiodarone
	M	Age, gender, diuretics, eGFR, peripheral arterial disease
Smith et al[53]	M	Age, gender, NYHA, diuretics, ACE inhibitor, renal failure
Stabile et al[54]	M	Model (used for LVEF, NYHA, CRT-D): LVEF, NYHA, CRT-D, at least one appropriate VT/VF
Stockburger et al[41]	M	Gender, programming arm, ischemic aetiology of cardiomyopathy, diabetes, heart rate, age, systolic blood pressure, LVEF, NYHA
Suleiman et al[27]	M	Age, sex, diabetes, history of atrial fibrillation/flutter, CAD, beta-blockers, NYHA, QRS, LVEF, prevention type
Weeke et al[21]	AS	Sex, age, QRS, LVEF, type of device, history of PCI, history of CABG
	M	Sex, age, QRS, LVEF, type of device, history of PCI, history of CABG, appropriate shock, inappropriate shock, appropriate therapy, inappropriate therapy (during the follow-up)
Wijers et al[55]	AS	LVEF, gender, ICM
	M	Gender, LVEF, QRS, GFR
Yung et al[24]	AS	Age, gender, ischemic cardiomyopathy, NYHA, syncope, peripheral vascular disease, chronic lung disease, current smoker, GFR, QRS, left atrial size, ACEi/ARB, loop diuretics
	M	Age, gender, NYHA, syncope, peripheral vascular disease, GFR, left atrial size, ACEi/ARB, loop diuretics

**Fig 2:**
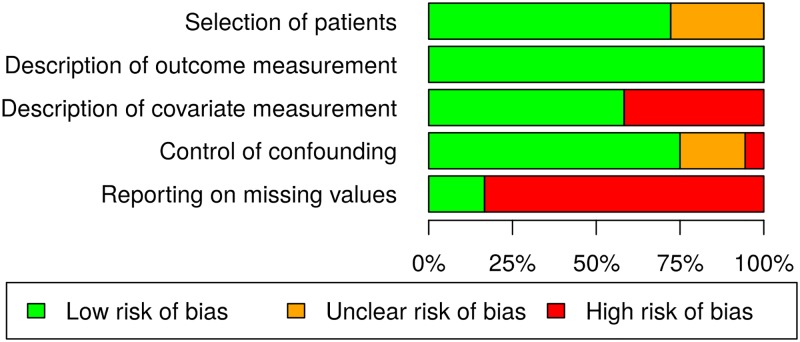
Visualization of the risk of bias assessments of the 36 identified studies. The unclear risk of bias regarding patients‘ selection is due to secondary analyses of a randomized controlled trial and studies with some specific inclusion criteria on the consecutive patients. In case of the missing values and the measurement of covariates, a high risk is associated with omitted reporting. The risk evaluation regarding confounders reflects the number of studies supplying only univariate results (high risk), both univariate and multivariate results (unclear risk), and only multivariate results (low risk) for our analyses.

Based on the assessment of the risk of bias we did not exclude any study from further analysis. A summary of the risk of bias assessments is provided in Fig 2.

### Device programming parameter

The programming of the ICD device can affect the occurrence of adequate therapy. Therefore, is desirable that the studies to be included used at least broadly speaking comparable device parameters. The programming parameters of each individual study are listed in Table 3. Not all studies have given information about programming, but those who are known are compatible with a high-rate cut-off therapy as defined in the MADIT-RIT population (38).

**Table 3:** Device programming parameters as given in the studies (n/a = not available; bpm = beats per minute; ATP = antitachycardic pacing).

Study	VT 1	VT 2	VF
Bilchick et al[35]	n/a	n/a	n/a
Biton et al[37]	n/a	170–199 bpm (ATP+shock)	>200 bpm (ATP during charge)
Campbell et al[42]	n/a	n/a	n/a
Demirel et al[46]	170–200 bpm (monitor only)	200–230 bpm (ATP+shock)	>230 bpm (ATP during charge)
Dichtl et al[44]	n/a	n/a	n/a
Fauchier et al[23]	n/a	n/a	n/a
Fernandez-Cisnal et al[28]	n/a	n/a	200 bpm
Gatzoulis et al[47]	n/a	160 bpm (ATP+shock)	200 bpm (no ATP)
Gigli et al[45]	n/a	n/a	n/a
Hage et al	n/a	n/a	n/a
Hager et al[31]	n/a	n/a	n/a
Konstantino et al, IMAJ[26]	n/a	n/a	n/a
Konstantino et al, ICE[43]	n/a	n/a	n/a
Kraaier et al[48]	n/a	n/a	n/a
Kutyifa et al[38]	n/a	170–199 bpm (ATP+shock)	200 bpm (ATP during charge)
	n/a	170–199 bpm (monitor)	200 bpm (ATP during charge)
	170–199 bpm (ATP+shock)	200–249 bpm (ATP+ shock)	>250 bpm (ATP during charge)
Lee et al[25]	n/a	n/a	n/a
Levine et al[32]	n/a	n/a	n/a
Maciag et al[49]	Up to 200 bpm	200–249 bpm	>250 bpm
Masoudi et al	n/a	n/a	n/a
Ng et al[50]	n/a	n/a	n/a
Nombela-Franco et al[51]	n/a	n/a	n/a
Providência et al[22]	n/a	n/a	n/a
Rodriguez-Mañero et al[29]	n/a	n/a	n/a
Ruwald et al[39]	n/a	170–199 bpm (ATP+shock)	200 bpm (ATP during charge)
	n/a	170–199 bpm (monitor)	200 bpm (ATP during charge)
	170–199 bpm (ATP+shock)	200–249 bpm (ATP+ shock)	>250 bpm (ATP during charge)
Sedláček et al[40]	n/a	170–199 bpm (ATP+shock)	200 bpm (ATP during charge)
	n/a	170–199 bpm (monitor)	200 bpm (ATP during charge)
	170–199 bpm (ATP+shock)	200–249 bpm (ATP+ shock)	>250 bpm (ATP during charge)
Seegers et al[52]	n/a	>170 bpm (ATP+shock)	>210–230 bpm (ATP during charge)
Smith et al[53]	n/a	>170 bpm (ATP+shock)	>200–220 bpm (ATP during charge)
Stabile et al[54]	n/a	160–200 bpm (ATP+shock)	>200 bpm (no ATP)
Stockburger et al[41]	n/a	170–199 bpm (ATP+shock)	200 bpm (ATP during charge)
	n/a	170–199 bpm (monitor)	200 bpm (ATP during charge)
	170–199 bpm (ATP+shock)	200–249 bpm (ATP+ shock)	>250 bpm (ATP during charge)
Suleiman et al[27]	n/a	n/a	n/a
Weeke et al[21]	n/a	n/a	n/a
Wijers et al[55]	n/a	n/a	n/a
Yung et al[24]	n/a	n/a	n/a

### Results of the meta-analyses

Regarding first inappropriate shock, for most of the considered factors none or just one published result was identified by our search. Therefore we do not report any meta-analyses for this endpoint. Thus four identified studies do not enter any of the following analyses (Biton, Chen, Fernandez-Cisnal, Kutyifa). An overview of the pooled hazard ratios reflecting effects of the considered factors on the other two end-points of interest is shown in Table 4. Presented are the number of studies pooled, the number of patients in the analysis and the pooled effects obtained by two different approaches as described in section Methods. We note that the standard, but possibly over-optimistic, method employing the DerSimonian-Laird estimator of the heterogeneity parameter and normal quantiles by construction of CIs (see also Methods) is reported mainly for comparison. A full account of all found literature results can be found in the following subsections devoted to the individual factors.

**Table 4:** Overview of pooled HRs regarding the end-points of interest and the considered factors. Shown is the number of studies, the number of patients and pooled HRs with their 95% CIs or credibility intervals (if Bayesian approach is applied) constructed as described in the section Methods. DL stands for the DerSimonian-Laird estimator of heterogeneity and normal quantiles used in the procedure and Bayes refers to the Bayesian procedure with half-normal prior (scale 0.5) for the heterogeneity parameter (between-study standard deviation). Note that in cases when less than 2 studies were identified by our search, no pooling was done (denoted by ----- in the table).

Studies/Patients Method: HR, 95% CI	First appropriate shock		All-cause mortality
	Instantaneous risks (Cox PH model)	Cumulative incidences (Fine & Gray model)	
Age at implantation, per 10 years	2/1054]	2/4077	11/4979
	Bayes: 0.97, [0.59, 1.60]	Bayes: 0.82, [0.51, 1.31]	Bayes: 1.42, [1.27, 1.58]
	DL: 0.96, [0.85, 1.09	DL: 0.82, [0.74, 0.91]	DL: 1.41, [1.29, 1.53]
LVEF	2/2162	-----	5/2923
	LVEF ≤ 25% vs LVEF ≥25%		Per 5% decrease
	Bayes: 1.28, [0.66, 2.68]		Bayes: 1.21, [1.10, 1.33]
	DL: 1.26, [0.89, 1.78]		DL: 1.21, [1.14, 1.29]
NYHA >II	-----	-----	10/30373
			Bayes: 1.71, [1.35, 2.22]
			DL: 1.72, [1.39, 2.12]
CRT-D	-----	-----	3/660
			Bayes: 0.95, [0.56, 1.65]
			DL: 0.94, [0.68, 1.30]
ICM	2/975	-----	9/11017
	Bayes: 1.97, [0.87, 5.53]		Bayes: 1.37, [1.06, 1.72]
	DL: 2.22, [0.83, 5.93]		DL: 1.37, [1.14, 1.66]
AF/AT	-----	-----	7/26048
			Bayes: 1.31, [1.08, 1.72]
			DL: 1.32, [1.13, 1.54]
Diabetes	-----	-----	7/20682Bayes: 1.44, [1.20, 1.82]DL: 1.41, [1.34, 1.49]
eGFR ≤ 60 mL/min/1.73m^2^	-----	-----	7/ 7752]
			Bayes: 2.30, [1.85, 2.85
			DL: 2.30, [1.97, 2.69]
Amiodarone	-----	2/4077 too heterogeneous to be pooled	3/18720
			Bayes: 1.22, [0.79, 2.30]
			DL: 1.27, [0.90, 1.80]
Beta-blockers	-----	-----	5/19743]
			Bayes: 0.73, [0.40, 1.11
			DL: 0.69, [0.45, 1.04]
Diuretics	-----	-----	5/23850
			Bayes: 1.53, [1.11, 2.35]
			DL: 1.55, [1.20, 2.00]

### Age at implantation

#### First appropriate shock

**Fig 3:**
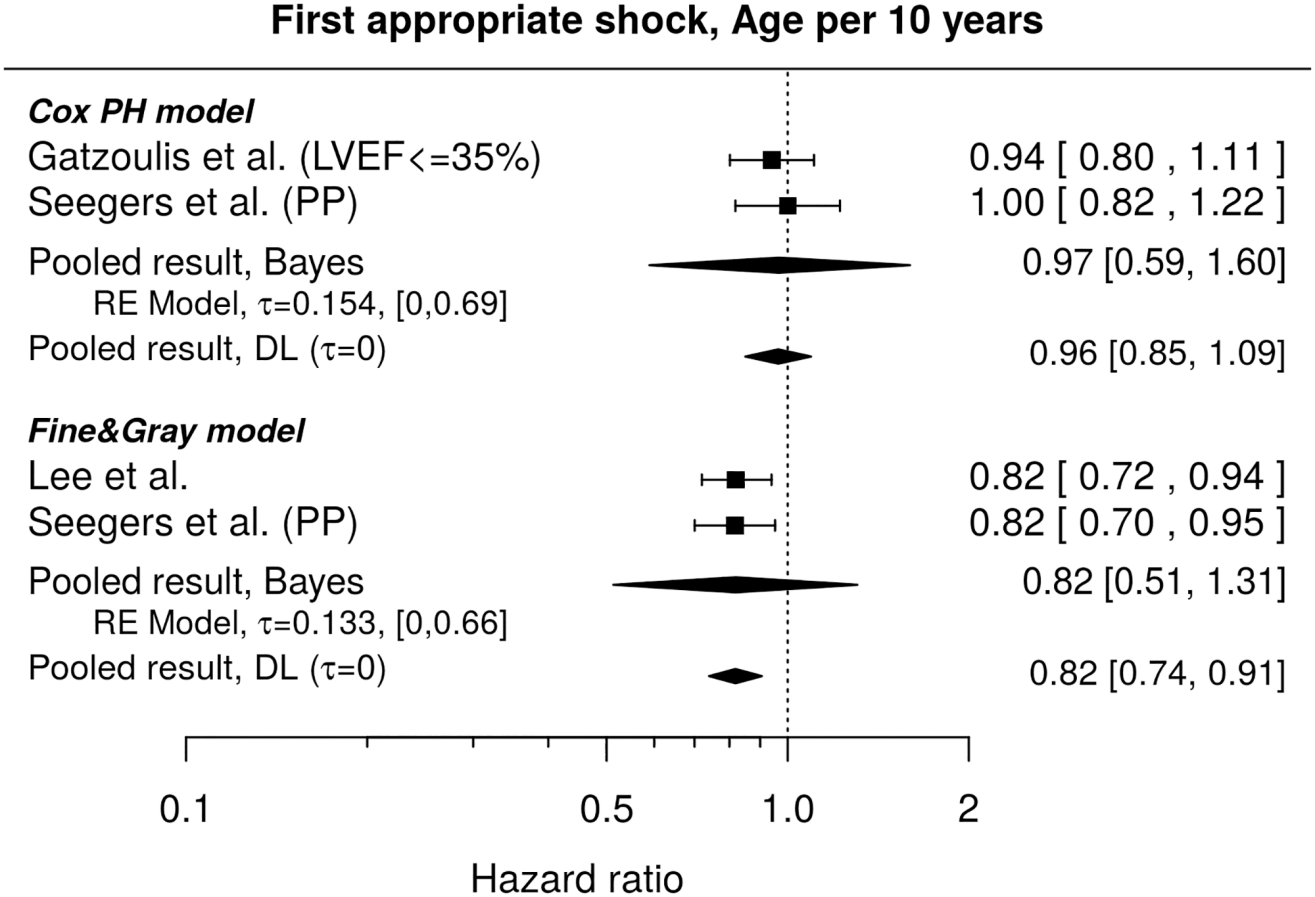
Forest plot showing pooled hazard ratios for the effect of age at implantation on instantaneous risk (Cox PH model) and cumulative incidence (Fine & Gray model) of the first appropriate shocks. HR of 1 corresponds to no age effect. PP indicates re-analysis of the primary prevention subgroup. Reported are the pooled results obtained by the Bayesian (Bayes) and the standard (DL) approach. DL stands for DerSimonian-Laird (for details see the section Methods).

The results found in literature[47,52] do not show a statistically significant influence of age on the instantaneous risk of the first appropriate shock (pooled HR per 10 years: 0.97, 95% credible interval [0.59, 1.60]), see Fig 3. The shown HRs agree with those reported by Weeke et al.[21], who did not find any significant difference when comparing age groups 65–74 and ≥75 with patients younger than 65 years (HRs and 95% CIs: 0.88 [0.6, 1.31] and 1.1 [0.64, 1.9], respectively).

Regarding the comparison of cumulative incidences, the HRs as shown in Fig 3 [25,52] suggest a lower cumulative incidence of the first appropriate shocks for older patients, nevertheless, the pooled effect (HR per 10 years 0.82) is accompanied by a credibility interval well overlapping 1 (95% credible interval [0.51, 1.31]), which is due to the small number of studies bringing in not much information on the between-study variance, and only a mild prior information on the heterogeneity used. It is to note that when age categories (18–49, 50–59, 60–69, 70–79, ≥80) were considered in a population closely related to Lee et al.[25], no statistically significant differences between the cumulative incidences in the single groups as compared to the youngest group were observed[24].

#### All-cause mortality

**Fig 4:**
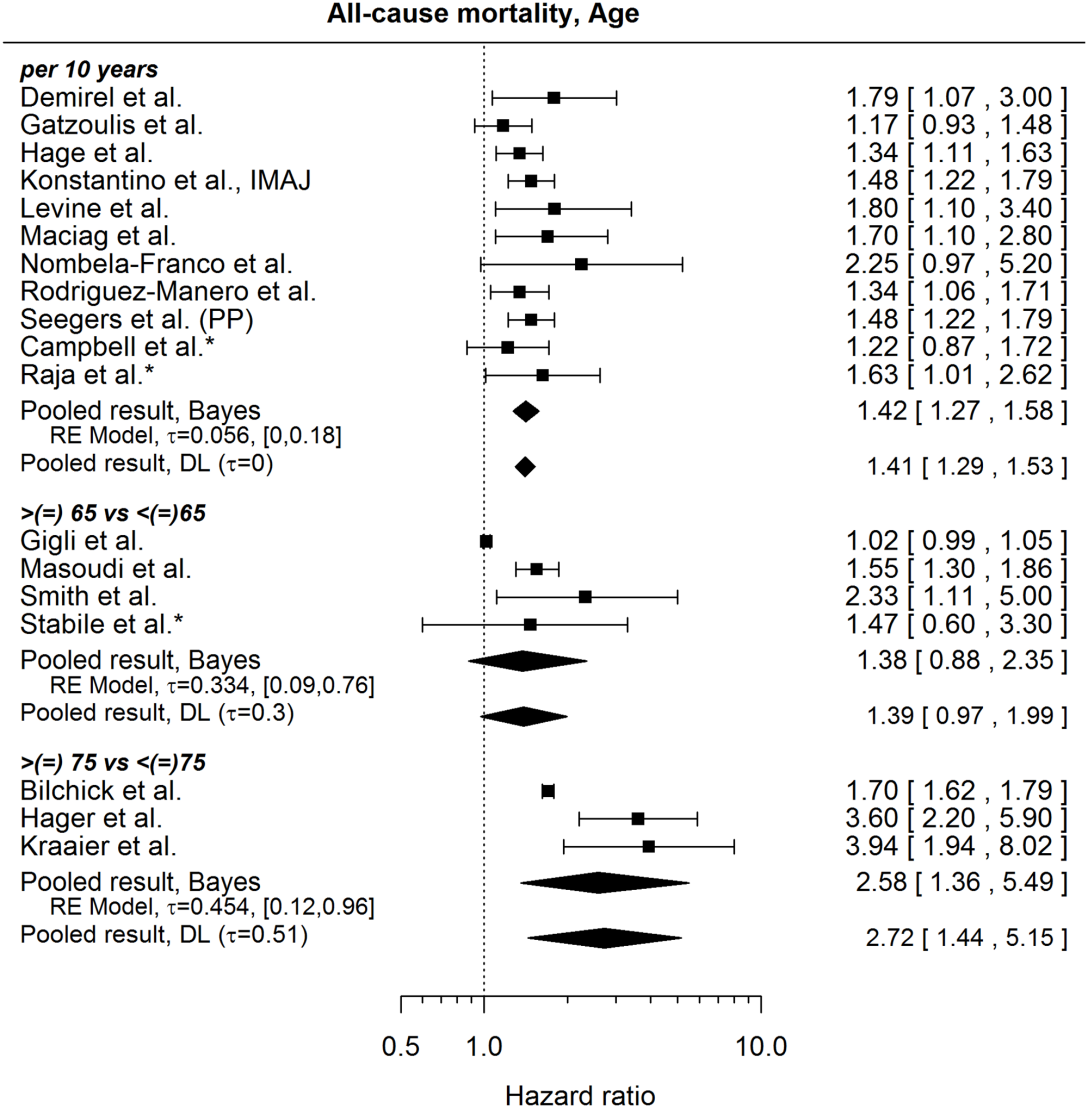
Forest plot showing pooled hazard ratios for the effect of age at implantation on the risk of death. Univariable models are denoted with *. HR of 1 corresponds to no age effect. Reported are the pooled results obtained by the Bayesian approach (Bayes) and the standard (DL: DerSimonian-Laird) approach (for details see the section Methods).

The published results regarding age and all-cause mortality are rather homogeneous when age is considered as a continuous variable, see Fig 4 (pooled HR per 10-year increase in age 1.42, 95% credible interval [1.27, 1.58]) and show considerable heterogeneity when age subgroups are compared (here it is to note that the studies differ as to the inclusion of the boundary age to the reference group). In addition to the HRs shown in Fig 4, we found the following mixture of results (all but one showing, a higher risk of death with increasing age; Table 5):

**Table 5:** Overview of hazard ratios (HR) and confidence intervals (in brackets) regarding age (ref. group = reference group; n.a. = not available).

	Ref. group	Subgroup 1	Subgroup 2	Subgroup 3	Subgroup 4
Fauchier et al[23]	< 60 years	60–75 years:	>75 years:	n.a.	n.a.
		HR 1.43	HR 1.65		
		[1.14–1.8]	[1.22–2.22]		
Weeke et al[21]	<65 years	65–74 years:	≥75 years:	n.a.	n.a.
		HR 2.11	HR 2.9		
		[1.47–3.02]	[1.91–4.42]		
Suleiman et al[27]	≤65 years	66–75 years:	>75 years:	n.a.	n.a.
		HR 1.4	HR 3.02		
		[0.67–2.91]	[1.56–5.86]		
Yung et al[24]	18–49 years	50–59 years:	60–69 years:	70–79 years:	≥ 80 years:
		HR 1.45	HR 2.21	HR 2.46	HR 2.99
		[0.72–2.93]	[1.15–4.27]	[1.28–4.72]	[1.46–6.1]
Dichtl et al[44]	≤70 years	>70 years:	n.a.	n.a.	n.a.
		HR 0.61			
		[0.25–1.52]			

Apparently a type occurred in Konstantino et al.[43] where HR 2.4 for >75 vs ≤75years with 95% CI [0.5, 1.3] and p = 0.0008 was reported. If the p-value and the HR would be correct, then the CI should be [1.44, 4.00] which compares well with the results in Fig 4.

### Left ventricular ejection fraction

#### First appropriate shock

**Fig 5:**
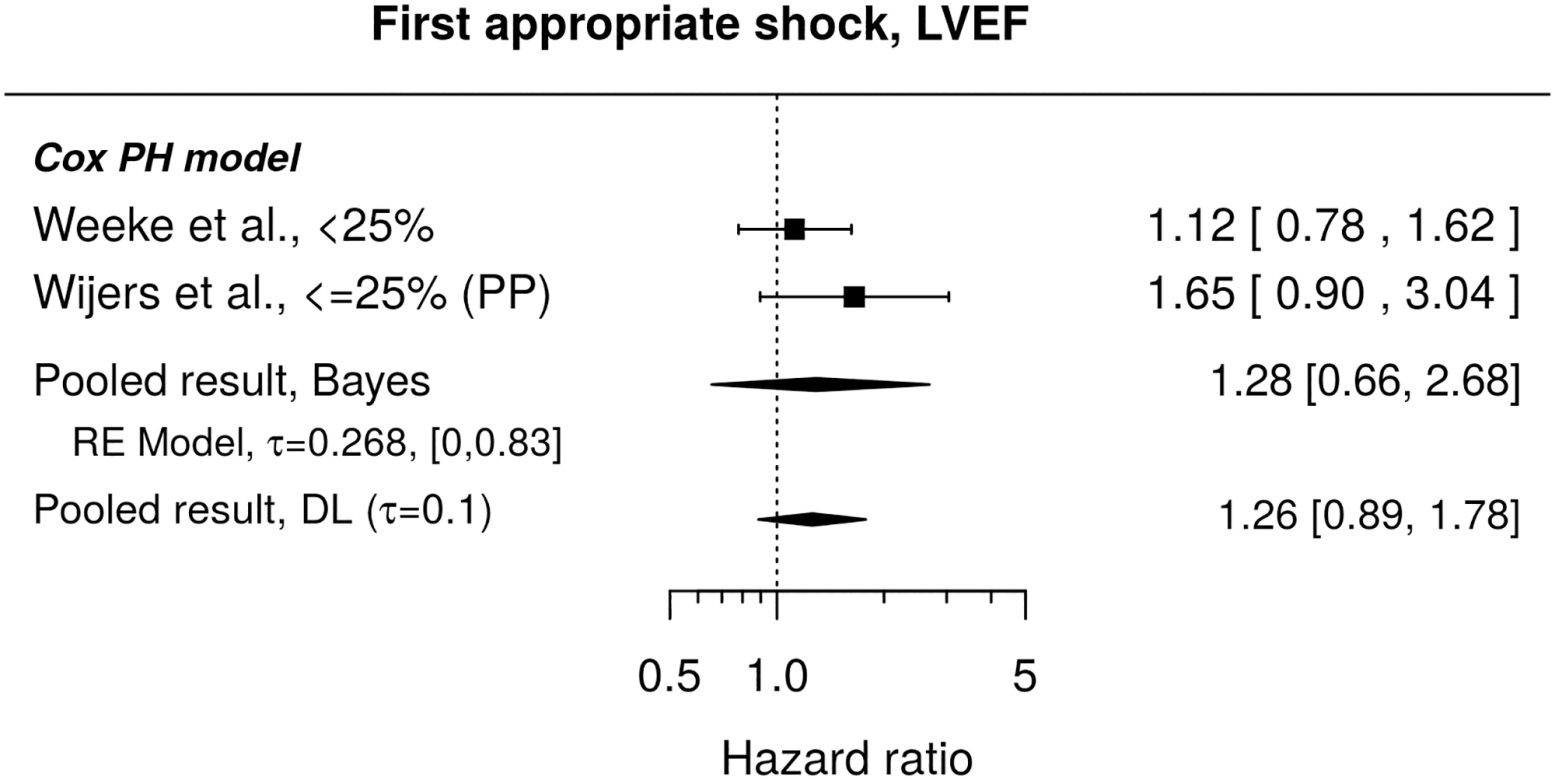
Forest plot showing pooled hazard ratio for the effect of LVEF at implantation on instantaneous risk (Cox PH model) of the first appropriate shock. HR of 1 corresponds to no LVEF effect. PP denotes re-analysis for primary prevention subgroup. Reported are the pooled results obtained by the Bayesian (Bayes) and the standard (DL) approach. DL stands for DerSimonian-Laird (for details see the section Methods).

Published results do not show a statistically significant influence of LVEF on the instantaneous risk of the first appropriate shock (pooled HR 1.28, 95% credible interval [0.66, 2.68]), see Fig 5. As to a direct comparison of cumulative incidences of the first appropriate shocks for different LVEF groups (i.e. HRs from Fine & Gray model), we found only one published study[25] and this reported no significant difference between patients with LVEF≤20% or those with LVEF 21–30% when compared to the group with LVEF 31–35% (unadjusted HRs and 95% CI: 1.56, [0.98, 2.50] and 1.21, [0.79, 1.85], respectively).

#### All-cause mortality

**Fig 6:**
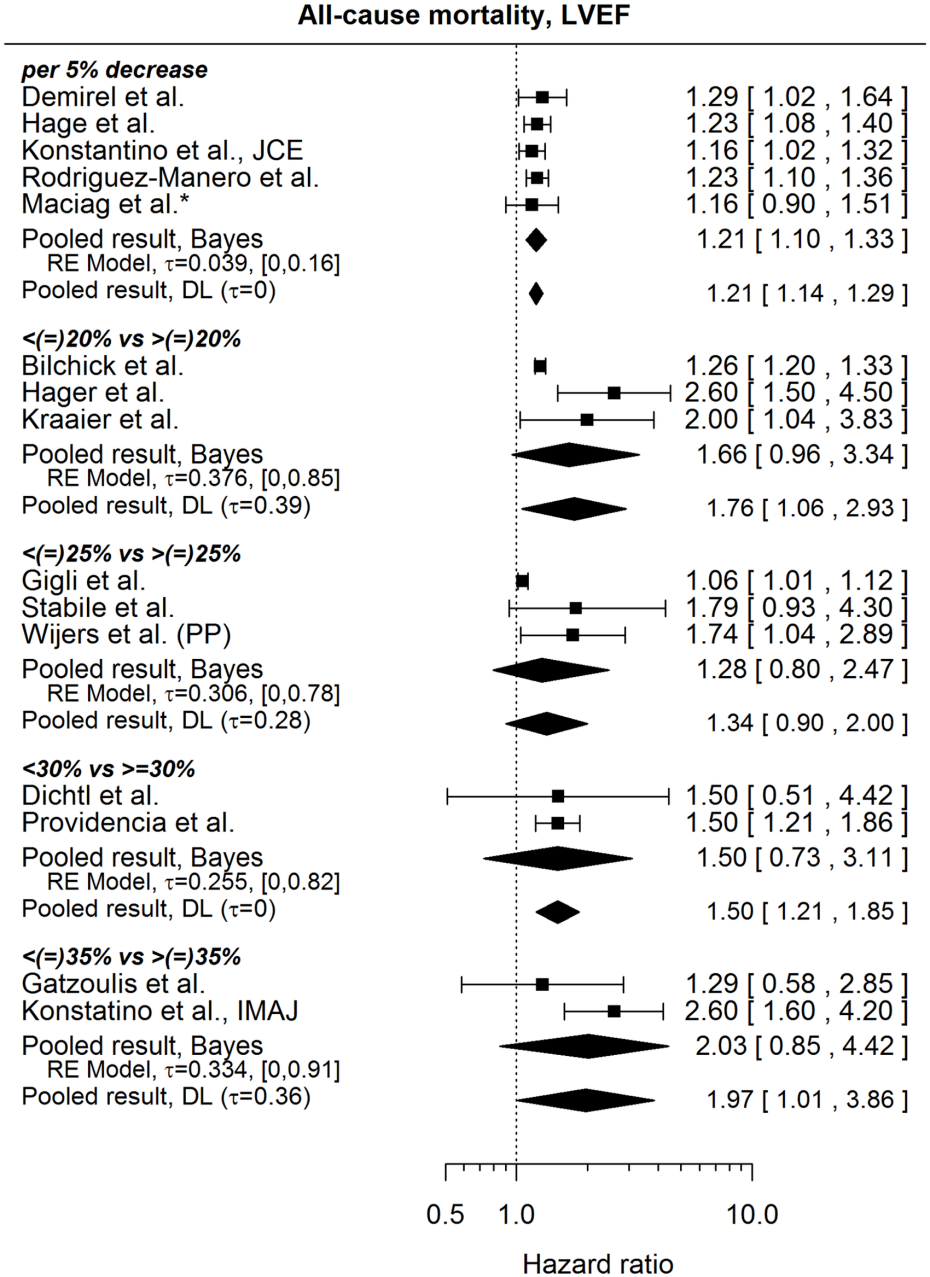
Forest plot showing pooled hazard ratios for the effect of LVEF at implantation on the risk of all-cause mortality. HRs coming from univariable models are denoted with *. HR of 1 corresponds to no LVEF effect. PP denotes re-analysis of primary prevention subgroup. Reported are the pooled results obtained by the Bayesian (Bayes) procedure and the standard (DL: DerSimonian-Laird) approach (for details see the section Methods).

Fig 6 shows the published results regarding the influence of LVEF on the risk of all-cause mortality. Unsurprisingly, the results mostly suggest a higher risk of death for patients with lower LVEF. The HRs from studies considering LVEF as a continuous variable are rather homogeneous (pooled HR per 5% decrease in LVEF 1.21, 95% credible interval: [1.10, 1.33]), whereas the HRs comparing certain categories of LVEF show, in general, more heterogeneity. It is also to note that the studies vary as to the inclusion of the border value of LVEF into the reference category. Results we were not able to include in our analysis consisted of an apparently erroneous HR (outside of the accompanying CI) by Campbell et al.[42] (HR of 0.91 per %, 95% CI: [0.96, 1.05]), a HR by Levine et al.[32] (2.35, [1.3, 3.7]) for which it was not clear in what way LVEF was considered in the model (continuous, or categories) and a result by Weeke et al.[21], where the HR was accompanied by a very asymmetric confidence interval on the log-scale, which makes it difficult to justify the calculation of the standard error of the HR and casts doubts on the correctness of the HR itself (HR of 1.26 for <25% vs ≥25%, 95% CI: [1.19,2.14]). The HR by Providencia et al.[22] in Fig 6 might be compared to the HR reported by Fauchier et al.[23], studying practically the same patient population (HR for LVEF<30% vs LVEF ≥ 30%: 1.67, 95% CI: [1.35, 2.04]).

### NYHA class

#### First appropriate shock

We found no studies regarding the influence of NYHA class on the instantaneous risk of the first appropriate shock. As to the comparison of cumulative incidences of first appropriate shocks between NYHA classes, two studies (based on the closely related patient population) reported no significant difference when patients with NYHA class III or IV were compared to those with NYHA class I or II[24,25] (HR 1.07, 95% CI: [0.81, 1.43] and HR 0.85, 95% CI: [0.66,1.1], respectively).

#### All-cause mortality

**Fig 7:**
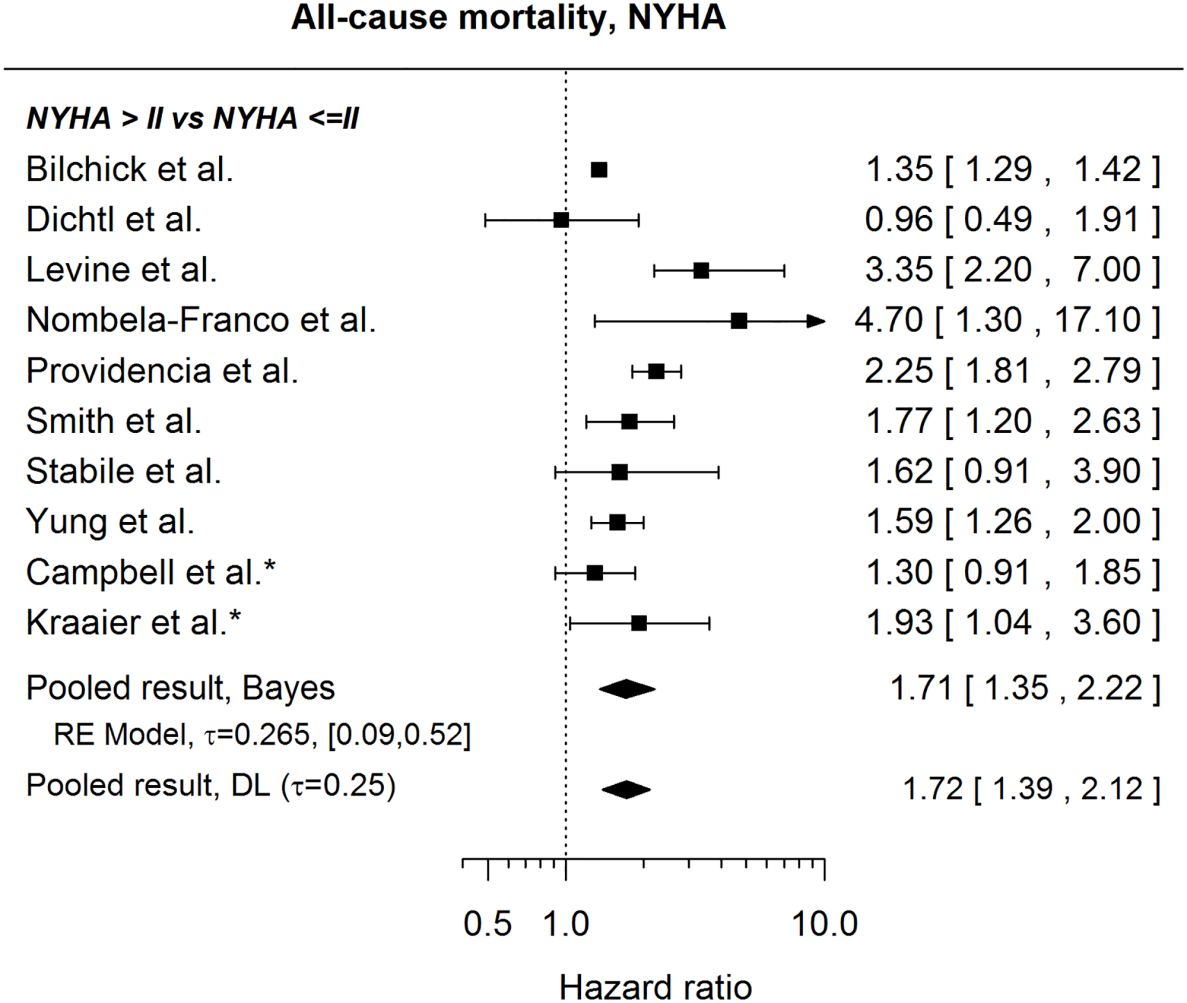
Forest plot showing pooled hazard ratio for the effect of NYHA class on the risk of all-cause mortality. HRs coming from univariable models are denoted with *. HR of 1 corresponds to no difference between NYHA classes >II and ≤ II. Reported are the pooled results obtained by the Bayesian (Bayes) and the standard (DL: DerSimonian-Laird) approach (for details see the section Methods).

The published results suggest that patients with NYHA class higher than II have a higher risk of death than patients with NYHA class I or II (pooled HR 1.71, 95% credible interval: [1.35, 2.22]), see Fig 7. It is to note, however, that not all NYHA classes were present in every studied population so that a HR for NYHA>II vs NYHA ≤II in Fig 7 can mean, III/IV vs I/II, or III vs I/II, or III/IV vs II. In addition to the results shown in Fig 7, several papers state a HR for a higher NYHA class without explicitly explaining what “higher” means. These HRs are stated in the Table 6.

**Table 6:** Overview of hazard ratios and confidence intervals regarding all-cause mortality and NYHA-class (* = univariate hazard ratio).

Study	Hazard ratio	Confidence interval
Demirel et al[46]*	1.71	1.02–2.87
Gatzoulis et al[47]	2.69	1.77–4.09
Maciag et al[49]	4.4	1.7–11.5
Ng et al[50]	1.96	1.15–3.33
Raja et al[34]*	4.3	1.9–9.5

The result by Providencia[22] in Fig 7 may be compared to the result by Fauchier[23] (HR 1.75, 95% CI: [1.42, 2.17]), since they studied the same patient population.

### CRT-D

Unlike in randomized controlled trials where patients eligible for CRT-D are randomized to CRT-D or ICD only and the benefit of CRT-D is evaluated, here we compare patients with CRT-D and those with ICD only in observational studies, where the decision, which device should be implanted, follows usually some guidelines. Thus the reported HRs do not evaluate a benefit of CRT-D over ICD alone, but compare the risk of the event of interest between two patient groups with certain indirectly defined sets of medical conditions.

#### First appropriate shock

Comparing CRT-D patients and those with single-chamber ICDs neither a significant difference in instantaneous risks of the first appropriate shock (HR 1.01, 95% CI: [0.62, 1.66][21], nor a significant difference in the cumulative incidences of this event (unadjusted HR 1.11, 95% CI: [0.81, 1.53][25] was found in the literature. Nevertheless, the reported results are too few to draw a general conclusion.

#### All-cause mortality

**Fig 8:**
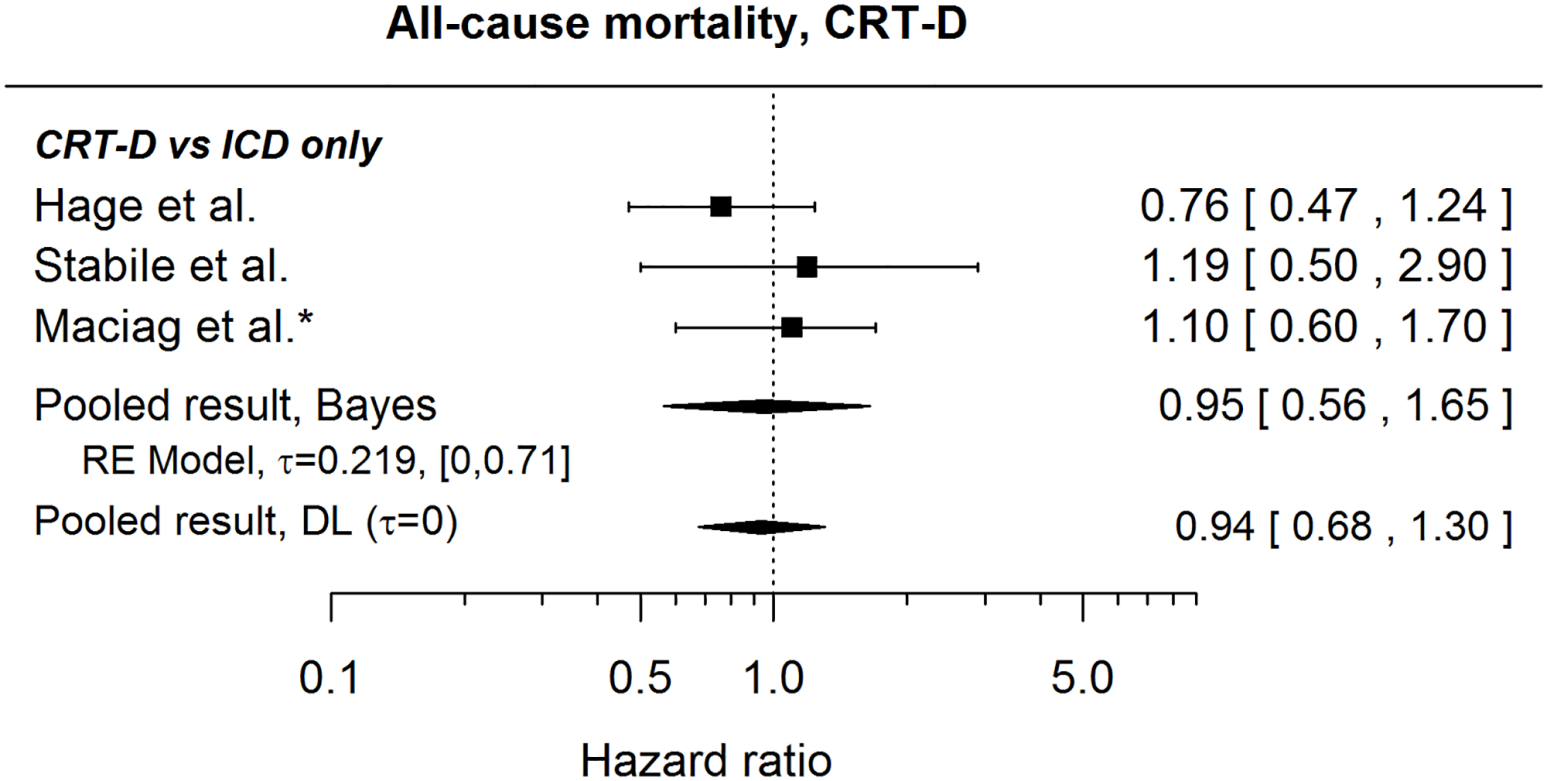
Forest plot showing pooled hazard ratio comparing the risk of all-cause mortality for CRT-D patients and those with ICD only. HRs coming from univariable models are denoted with *. HR of 1 corresponds to no difference between the groups. Reported are the pooled results obtained by Bayesian (Bayes) and the standard (DL: DerSimonian-Laird) approach (for details see the section Methods).

Fig 8 presents published results regarding a comparison of risk of death for CRT-D and ICD only patients (the studies do not specify percentages of single- and dual-chamber ICDs in the ICD only group) together with a pooled HR (0.95, 95% credible interval: [0.56, 1.65]) suggesting no difference in risks. This is in agreement with a statement in Ng et al.[50] claiming no difference between the groups (no HR given). Furthermore, no statistically significant difference was found by Weeke et al.[21] and Kraaier et al.[48] when comparing CRT-Ds with specifically single-chamber ICDs (HR 1.09, 95% CI: [0.72, 1.65] and unadjusted HR 1.25, 95% CI: [0.55, 2.84], respectively). In contrast, Stockburger et al.[41] found a significantly lower risk of death for CRT-D patients when compared with those with dual-chamber ICDs (HR 0.4, 95% CI: [0.23, 0.69]).

### Comorbidities

Here we focus on the effect of atrial fibrillation/tachyarrhythmia, diabetes and ischemic cardiomyopathy on the risk of the end-points.

#### First appropriate shock

**Fig 9:**
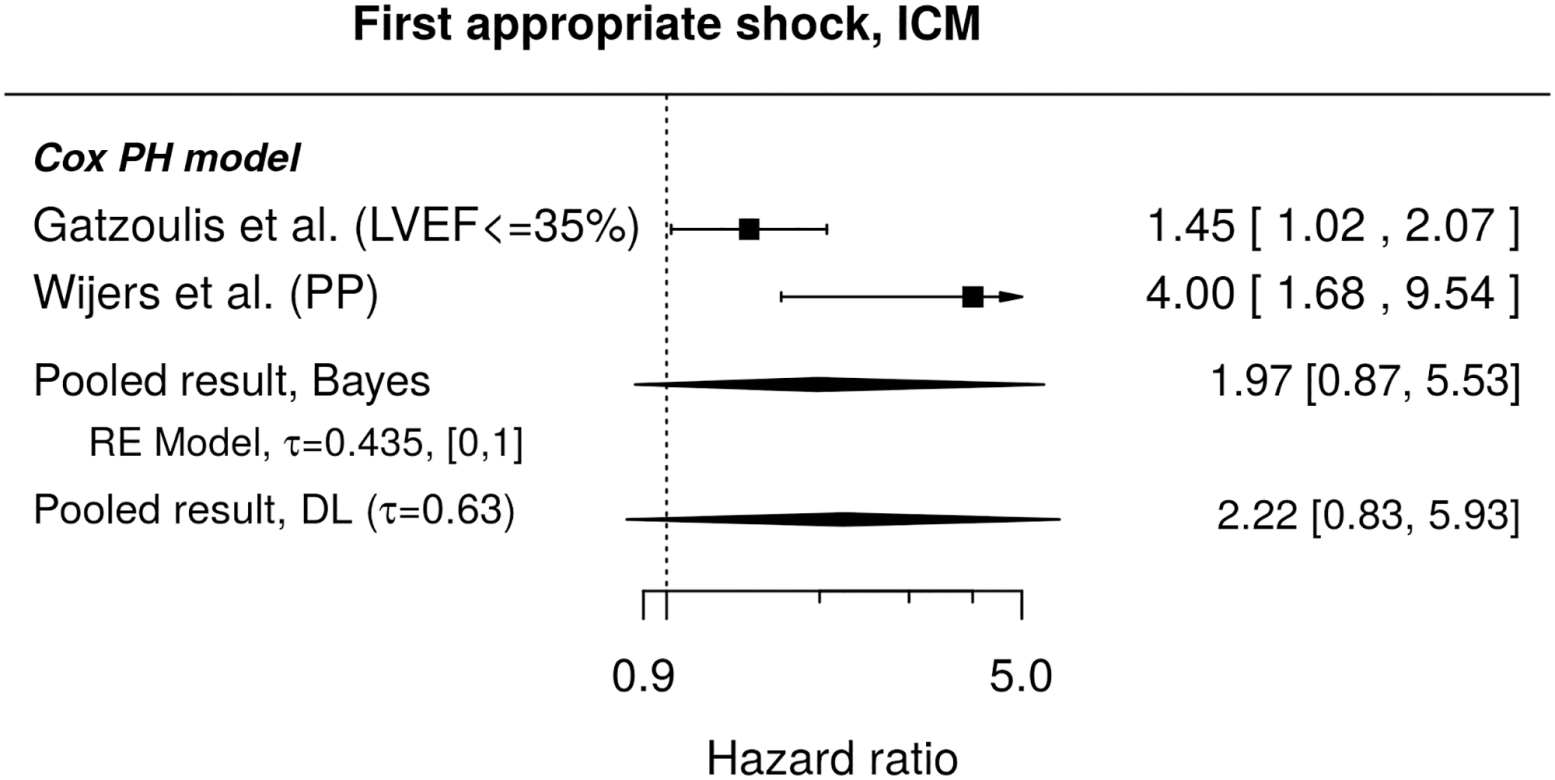
Forest plot showing pooled hazard ratio comparing instantaneous risks of the first appropriate shock for patients with ICM and those without this condition. HR of 1 corresponds to no difference between the groups. PP indicates a re-analysis for the primary prevention subgroup. Reported are the pooled results obtained by the Bayesian (Bayes) and the standard (DL) approach. DL stands for DerSimonian-Laird (for details see the section Methods).

Results regarding the chosen comorbidities were rather scarce in literature. Lee et al[25] observed a higher cumulative incidence of the first appropriate shocks in patients with prior or present atrial fibrillation (HR 1.61, 95% CI: [1.17, 2.21]). No effect of diabetes on the cumulative incidence was found in Lee et al.[25] (unadjusted HR 1.12, 95% CI: [0.84, 1.49]), but Ruwald et al.[39] report a higher instantaneous risk of the first appropriate shock for patients with diabetes (HR 1.62, 95% CI: [1.01, 2.61]). Results regarding ischemic cardiomyopathy and instantaneous risk of first appropriate shock are presented in Fig 9. The individual studies report higher risk associated with ischemic cardiomyopathy, however, the reported HRs are rather heterogeneous and the pooled effect is accompanied by a credible interval including 1. Regarding cumulative incidences, no significant difference associated with ischemic cardiomyopathy was found in the only study identified in our search[24], HR 0.81, 95% CI: [0.62, 1.05].

#### All-cause mortality

**Fig 10:**
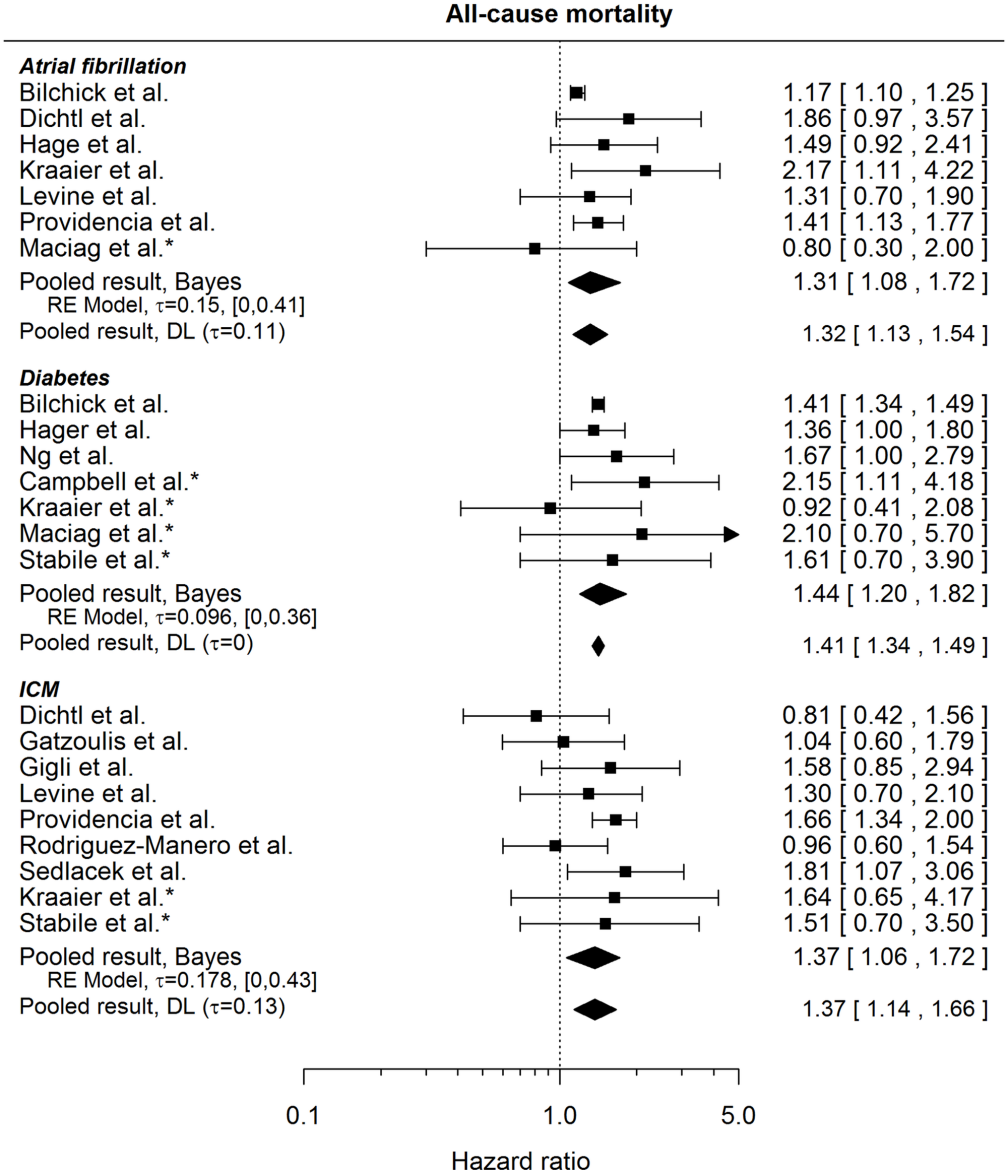
Forest plot showing pooled hazard ratios comparing the risk of all-cause mortality for patients with comorbidities and those without. HRs coming from univariable models are denoted with *. HR of 1 corresponds to no difference between the groups. Reported are the pooled results obtained by the Bayesian (Bayes) and the standard (DL: DerSimonian-Laird) approach (for details see the section Methods).

All 3 comorbidities seem to increase the risk of death (pooled effects 1.31, 95% credible interval: [1.08, 1.72], 1.44, 95% credible interval: [1.20, 1.82] and 1.37, 95% credible interval: [1.06, 1.72] for atrial fibrillation, diabetes and ischemic cardiomyopathy, respectively), see Fig 10. However, the definitions of the conditions are not always clearly described in the published studies; Kraaier et al.[48], Levine et al.[32], Providencia et al.[22] speak specifically about the history of AF, similarly as Fauchier et al.[23], whose result may be compared to that of Providencia et al.[22], since they are both based on the same patient population (HR 1.32, 95% CI: [1.08, 1.62]). The HRs for ischemic cardiomyopathy compare risk for patients with and without this condition. The latter may include patients with only non-ischemic cardiomyopathy, or patients with dilated cardiomyopathy, or a mixture of different other conditions[44]. Gigli et al[45] and Dichtl et al[44] use coronary artery disease (CAD) and ischemic cardiomyopathy interchangeably, Levine et al[32] give a HR for history of CAD, CAD is included in the definition of ICM by Providencia et al.[22], and Fauchier et al.[23] analysing the same population speak about CAD vs non-ischemic patients and give a HR of 1.32, 95% CI: [1.08, 1.61].

### Renal function

#### First appropriate shock

Regarding the first appropriate shock, Lee et al[25] report a higher cumulative incidence of the events for patients with increased creatinine levels (HR per mg/dl 1.21, 95% CI: [1.05, 1.39]). When almost the same population was studied by Yung et al.[24] regarding eGFR (calculated by Modification of Diet in Renal Disease formula), no significant differences for a comparison of subgroups were found. Namely, patients with eGFR 30–60 and <30 were compared to patients with GFR>60 (HR 1.13, 95% CI: [0.64, 2] and HR 1.2, 95% CI: [0.92, 1.57] respectively).

#### All-cause mortality

**Fig 11:**
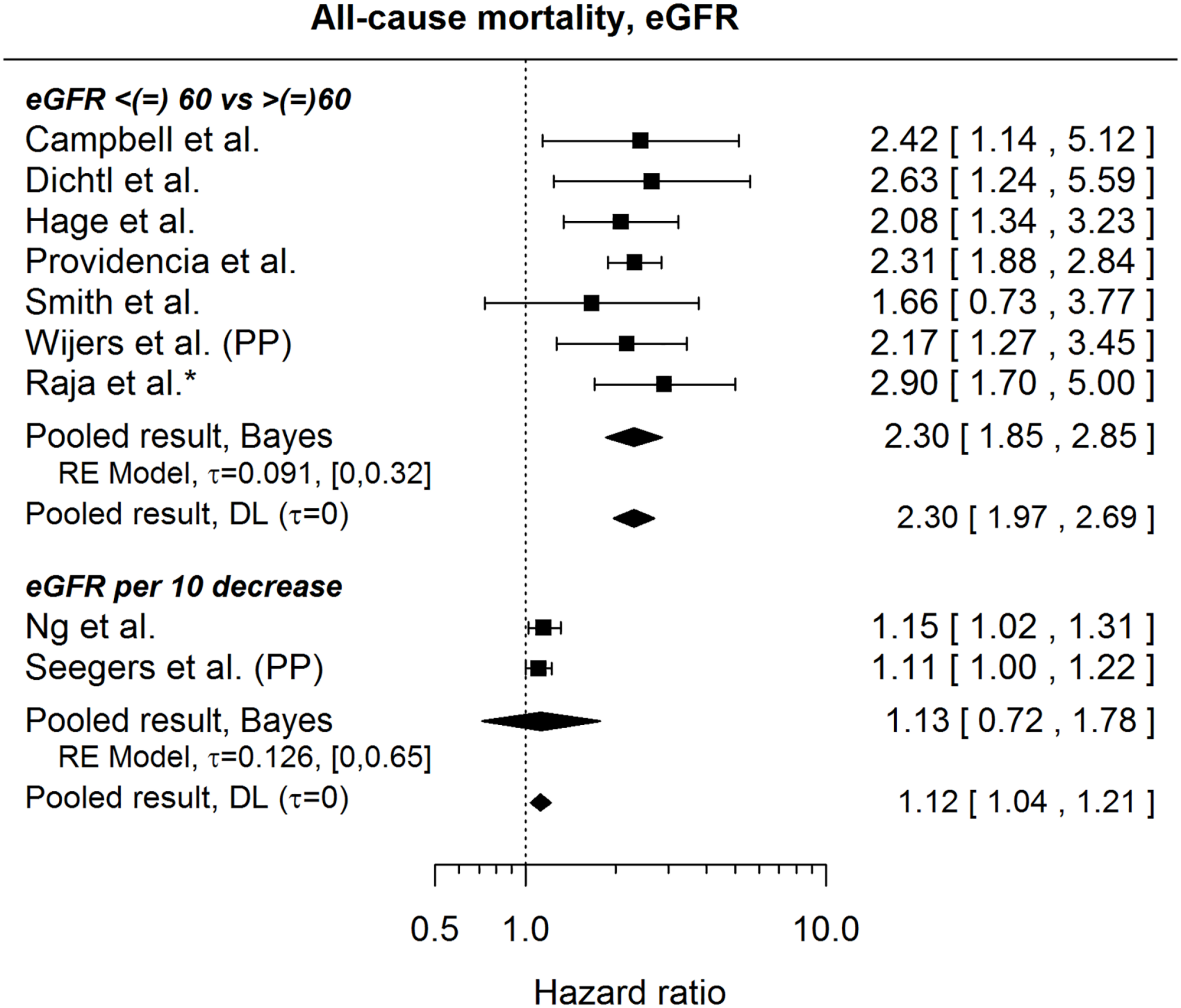
Forest plot showing pooled hazard ratios capturing the effect of a decreasing eGFR on the risk of all-cause mortality. HRs coming from univariable models are denoted with *. eGFR is measured in mL/min/1.73 m2 and calculated by the Cockroft-Gault formula (22) or the MDRD (Modification of Diet in Renal Disease) formula (all other studies). HR of 1 corresponds to no effect of decreasing eGFR. PP indicates re-analysis for primary prevention subgroup. Reported are the pooled results obtained by the Bayesian (Bayes) procedure and the standard (DL: DerSimonian-Laird) approach (for details see the section Methods).

Fig 11 summarizes HRs concerning the risk of death and eGFR levels (measured in mL/min/1.73m^2^). Apart from the results shown in Fig 11, Table 7 summarizes comparisons of different eGFR subgroups:

**Table 7:** Overview of hazard ratios and confidence intervals regarding all-cause mortality and renal function (eGFR = estimated glomerular filtration rate, HR = Hazard ratio, confidence intervals in brackets, n.a. = not available).

	Subgroup 1	Subgroup 2	Subgroup 3	Subgroup 4	Subgroup 5
Hager et al.[31]	eGFR >90 HR 1	eGFR: 60–89 HR 1.08 [0.6–2]	eGFR: 30–59 HR 1.37 [0.7–2.6]	eGFR: 15–29 HR 3.1 [1.5–6.3]	0–14 HR 10.2 [4.2–24.1]
Hess et al[36]	eGFR: >60 HR 1	eGFR: 30–60 HR 2.08 [1.99–2.18]	eGFR: < 30 HR 4.2 [3.92–4.5]	On dialysis HR 4.2 [4.46–5.17]	n.a.
Kraaier et al[48]	eGFR: >30 HR 1	eGFR: < 30 HR 3.14 CI: [0.96, 10.3]	n.a.	n.a.	n.a.
Yung et al[24]	eGFR: >60 HR 1	eGFR: 30–60 HR 2.04 CI: [1.57, 2.63]	eGFR: <30 HR 3.55 [2.45–5.13]	n.a.	n.a.

Furthermore, Bilchick et al.[35] refer to Chronic Kidney Disease (CKD) without giving a detailed definition and observe a higher risk of death for CKD patients (HR 2.28, 95% CI: [2.15, 2.41]). An increased risk of death was also found[43] for patients with chronic renal failure (HR 1.57, 95% CI: [1.09, 2.26]) and for patients with renal failure (unadjusted HR 2.4, 95% CI: [1, 6.1])[49]. Rodriguez-Mañero et al[29] found higher risk of death to be associated with increased creatinine levels (HR per mg/dl 1.66, 95% CI [1.36, 2.02]).

### Medication at implantation

Here we focus on the use of Amiodarone, beta-blockers and diuretics.

#### First appropriate shock

**Fig 12:**
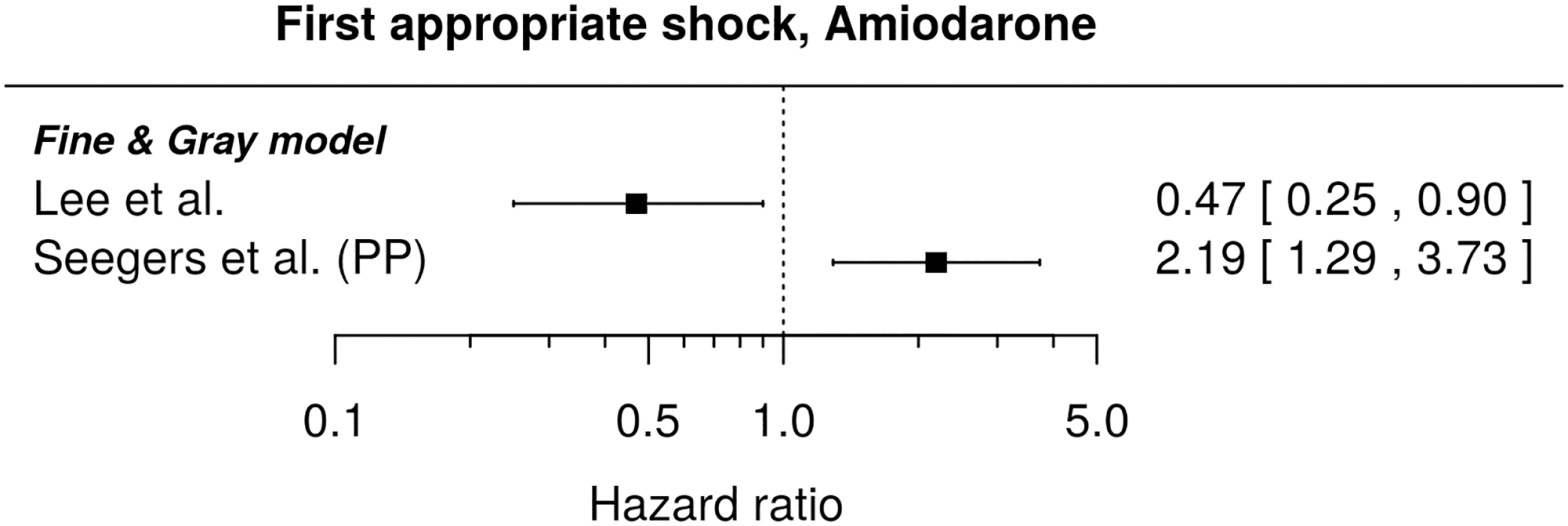
Forest plot showing hazard ratios comparing cumulative incidences of the first appropriate shock for patients with prescribed Amiodarone and those without. HR of 1 corresponds to no difference between the patient groups. PP indicates re-analysis for the primary prevention subgroup.

Regarding the instantaneous risk of the first appropriate shock and the chosen medication, we found only one result, which for the primary prevention subgroup states a higher risk for those with prescribed Amiodarone (HR 1.96, 95% CI: [1.24, 3.11])[52]. Regarding the comparison of cumulative incidences, Fig 12 shows the available results for Amiodarone. The published HRs are very heterogeneous (showing in opposite directions), so that we did not attempt their pooling. For patients with and without prescribed beta-blockers and loop diuretics, no significant difference in cumulative incidences was described (unadjusted HRs and 95% CIs: 1.27, [0.77, 2.08] and 1.36, [1.00, 1.84], respectively)[25]. The latter HR can be compared to the result by Yung et al.[24] based on a multivariable model and almost the same patient population (HR 1.15, 95% CI: [0.88, 1.5]).

#### All-cause mortality

**Fig 13:**
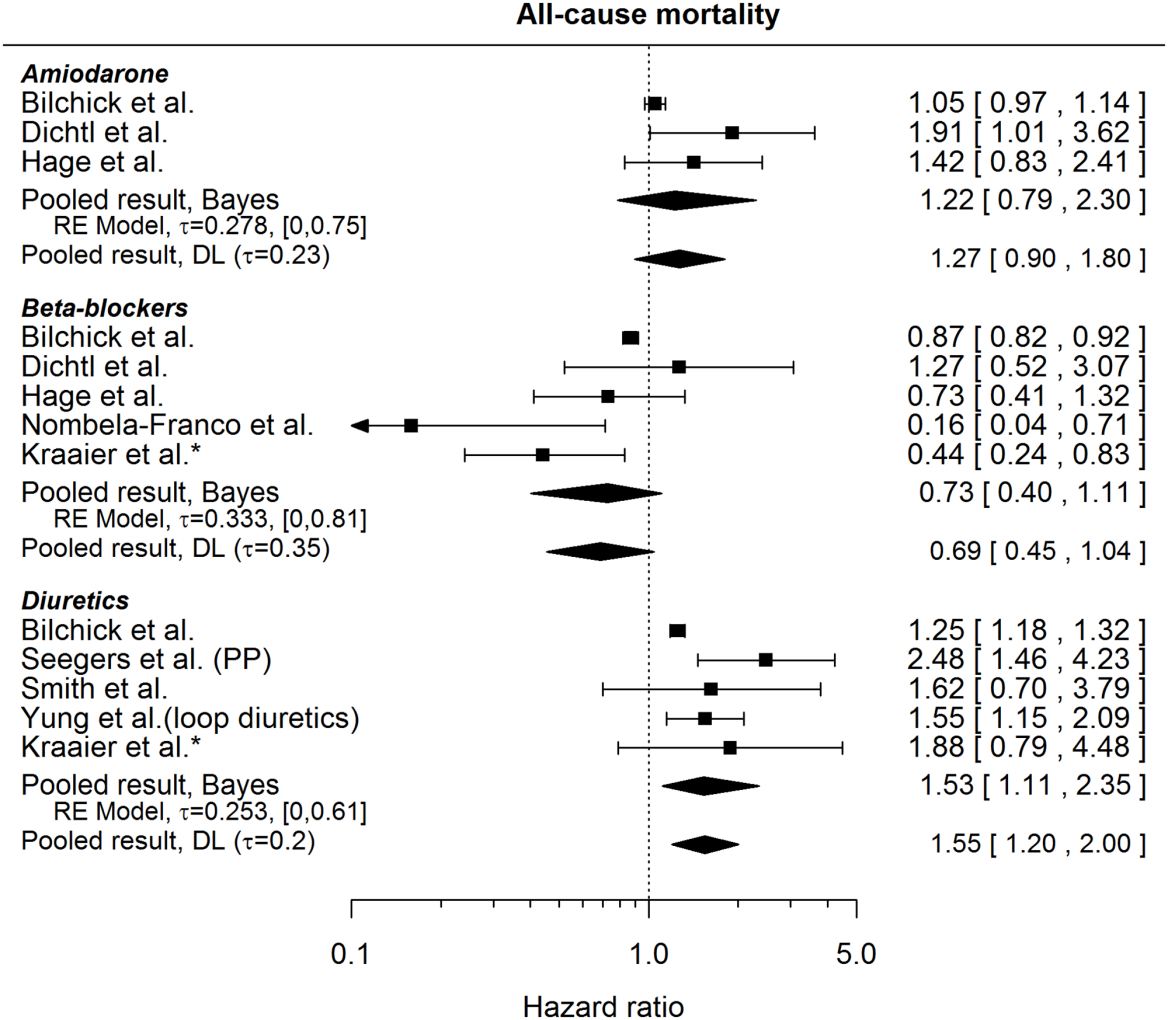
Forest plot showing pooled hazard ratios comparing the risk of all-cause mortality for patients with medication prescribed at baseline and those without. HRs coming from univariable models are denoted with *. HR of 1 corresponds to no difference between the groups. PP indicates a re-analysis for the primary prevention subgroup. Reported are the pooled results obtained by the Bayesian (Bayes) and the standard (DL: DerSimonian-Laird) approach (for details see the section Methods).

HRs comparing the risk of death for patients with prescribed medication and those without as found in the literature are presented in Fig 13. A clearly higher risk of death was found to be associated only with diuretics (pooled HR 1.53, 95% CI: [1.11, 2.35]). Not shown in the Fig 13 is the result regarding Amiodarone as stated by Levine et al.[32], since the CI is rather asymmetric on the log-scale, which casts doubts on the calculation of the standard error of the HR and the correctness of the HR itself (HR 0.72, 95% CI [0.50, 1.90]).

## 5 Discussion

In this meta-analysis we sought to determine risk factors for the occurrence of appropriate shocks and all-cause mortality in patients with a primary prophylactic ICD indication. While we were able to describe several risk factors for all-cause mortality, no conclusive risk factors for the occurrence of appropriate shocks were identified. We observed that the occurrence of appropriate shocks was rarely defined as an endpoint in the examined studies. One possible explanation might be the deliberate exclusion of all studies, which solely reported “appropriate therapy” as an endpoint, rather than distinguishing between appropriate shock and appropriate ATP. The authors feel, however, that this differentiation is necessary as the administration of one appropriate shock is regarded as equivalent to a survived SCD. In this respect, it has already been shown that therapy reduction programming is not associated with an increased risk of SCD[56]. Inclusion of appropriate ATP administration as an endpoint may therefore overestimate the effect of ICD therapy on the patients’ survival. We are aware that the occurrence of ICD-discharges is correlated to the device parameters. Unfortunately, not all studies reported these settings. However, the reported ones indicate that a reasonable approach to ICD-therapy is broadly used. In this context, appropriate ICD discharges might be the most suitable parameter to estimate the rate of prevented SCD.

Our results indicate that older patients receive fewer shocks than younger ones (HR 0.82 in the Fine and Gray model). This effect was significant when using the standard DerSimonian and Laird procedure. These results are fully consistent with the recently published DANISH-ICD trial[57]. As expected, higher age was associated with an increased risk of all-cause mortality. Following these findings, elderly patients are not expected to experience similar benefit of ICD treatment as younger patients do. Further studies must clarify whether this should be considered in decision making for ICD therapy. Ischemic cardiomyopathy as an underlying cardiac disease was found to be a conclusive risk factor for the occurrence of appropriate shocks in most individual studies. Due to the heterogeneity of the HRs, the pooled results (HR 1.97) turned out as non-significant, indicating, however, that the risk of appropriate shocks may be higher in ICM patients compared to other PP-ICD candidates. In contrast to appropriate shock risk, all-cause mortality is predictable by several independent factors. The association of LVEF and NYHA class with all-cause mortality may be of particular note. Our data confirm that an impaired left ventricular function is associated with an increased risk of death. In conclusion, patients with a PP-ICD indication are at an elevated risk of death of any cause irrespective of their risk of SCD. LVEF as a single factor is not sufficient to identify patients with benefit from ICD therapy. This observation is supported by the recent DANISH-trial[57]. In this prospective randomized controlled trial, patients with non-ischemic cardiomyopathy were randomly assigned to receive ICD/CRT-D therapy or conservative treatment. While the rate of SCD in the ICD-treated group was lower, the total mortality rate was not statistically significantly decreased by ICD-therapy.

There are already various risk scores for the calculation of the individual mortality risk of ICD patients in the literature[7,58]. The scores consist of a combination of different clinical factors, most of which are confirmed by the results of our study. However, in order to assess the individuals’ benefit of ICD therapy, risk scores for the occurrence of appropriate shocks comparable to the mortality risk scores should be evaluated in future research.

From a methodological point of view, our consequent presentation of pooled results obtained by two different methods for random-effects meta-analysis, namely the Bayesian approach and standard methodology based on the DerSimonian-Laird estimator, allows for a comparison of their performance across meta-analyses combining 2 to 11 available studies. With the notable exception of very few studies (say 2 to 3) to be combined the pooled hazard ratios do not differ considerably between the two approaches. In case of very few studies the accompanying credible/confidence intervals are often of a rather different length. The Bayesian procedure tends to yield wider intervals, which often prevents the claim of a statistically significant effect. In simulation studies [15,16,18] it has been demonstrated that the DerSimonian-Laird approach yields confidence intervals that are simply to short. This has mainly to reasons. Firstly, the between-study heterogeneity is often underestimated with only very few studies. Secondly, the uncertainty in estimating the between-study heterogeneity is large and not accounted for in the construction of the confidence intervals. A meta-analysis of individual patient data (IPD) would have allowed further insights into the importance of the risk factors and their combinations. Here we limited our investigations to the use of published aggregated data as and IPD meta-analysis would have been beyond the scope of this study. Also access to the various cohorts might not have been possible as their data is not publicly available. However, we believe that the use of the Bayesian methods yielded in robust analyses by adequately reflecting heterogeneity between the cohorts and their analysis methods.

The overall median start of enrollment was 2003, till the median end of enrollment of 2010. The median of this time period was calculated to be 2007. Based on this time frame of data collection, the given values, and time of publication of results, we believe we matched the criteria of a „contemporary population“.

In conclusion, the identification of patients with the most benefit from ICD therapy is still challenging. Risk factors for the occurrence of appropriate shocks have not yet been identified reproducibly in the literature. LVEF clearly predicts all-cause mortality, whereas its effect on the risk of appropriate shocks is ambiguous. Observational prospective studies, such as the EU-CERT-ICD (NCT 02064192) are designed to evaluate valid risk factors for the occurrence of appropriate shocks in PP-ICD-patients. The results will provide a valuable contribution to the scientific discourse and future ICD-therapy.

## Supporting information

S1 Fig PRISMA checklist. PRISMA checklist of items to include when reporting a systematic review or meta-analysis.
(PDF)

## Abbreviations

AF: atrial fibrillation
CAD: coronary artery disease
CI: confidence interval
CKD: chronic kidney disease
CRT: cardiac-resynchronization therapy
GFR: Glomerular filtration rate
GFR: glomerular filtration rate
HR: hazard ratio
ICD: implantable cardioverter-defibrillator
ICM: ischemic cardiomyopathy
LVEF: left ventricular ejection fraction
NYHA class: New York Heart Association functional class
PH: proportional hazards
PP: Primary prevention
s.e.: standard error
